# Pathophysiological Roles of Abnormal Axon Initial Segments in Neurodevelopmental Disorders

**DOI:** 10.3390/cells10082110

**Published:** 2021-08-17

**Authors:** Masashi Fujitani, Yoshinori Otani, Hisao Miyajima

**Affiliations:** Department of Anatomy and Neuroscience, Faculty of Medicine, Shimane University, 89-1 Enya-cho, Izumo-shi 693-8501, Shimane, Japan; yotani@med.shimane-u.ac.jp (Y.O.); miyajima@med.shimane-u.ac.jp (H.M.)

**Keywords:** axon initial segment (AIS), action potential (AP), ankyrin-G, spectrins, plasticity, neurodevelopmental disorders (NDDs)

## Abstract

The 20–60 μm axon initial segment (AIS) is proximally located at the interface between the axon and cell body. AIS has characteristic molecular and structural properties regulated by the crucial protein, ankyrin-G. The AIS contains a high density of Na^+^ channels relative to the cell body, which allows low thresholds for the initiation of action potential (AP). Molecular and physiological studies have shown that the AIS is also a key domain for the control of neuronal excitability by homeostatic mechanisms. The AIS has high plasticity in normal developmental processes and pathological activities, such as injury, neurodegeneration, and neurodevelopmental disorders (NDDs). In the first half of this review, we provide an overview of the molecular, structural, and ion-channel characteristics of AIS, AIS regulation through axo-axonic synapses, and axo−glial interactions. In the second half, to understand the relationship between NDDs and AIS, we discuss the activity-dependent plasticity of AIS, the human mutation of AIS regulatory genes, and the pathophysiological role of an abnormal AIS in NDD model animals and patients. We propose that the AIS may provide a potentially valuable structural biomarker in response to abnormal network activity in vivo as well as a new treatment concept at the neural circuit level.

## 1. Introduction

**Figure 1:**
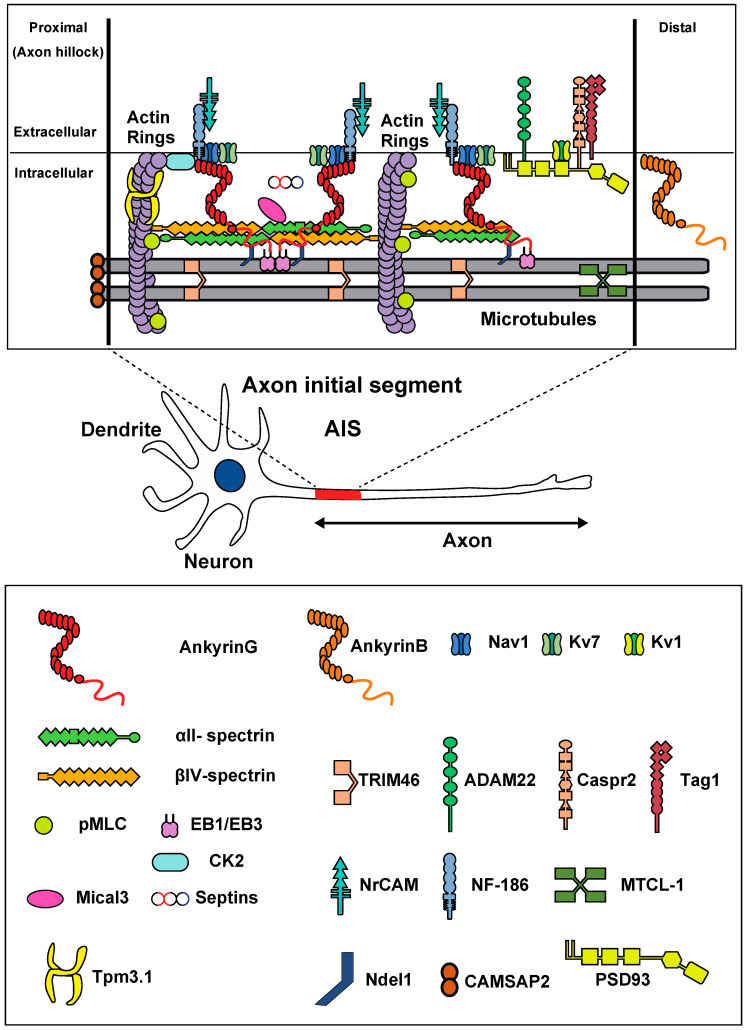
Molecular organization of the AIS, and diagram of a multipolar neuron. Membrane proteins (Nav/Kv7 channels, NF-186, NrCAM) are anchored by ankyrin-G (red). Ankyrin-G is inserted into the αII (green)/βIV (orange) spectrin tetramers. Actin rings (purple) are connected with spectrin tetramers. In the distal part, Kv1 channels, ADAM22, Tag1, and Caspr2 are present. Ankyrin-G binds to microtubules via EB1/EB3 proteins and Ndel1. Bundles of microtubules are crosslinked by TRIM46 and MTCL-1. Actin rings appear as twisted ropes possessing two long, intertwined actin filaments connected by a dense mesh of aligned spectrins. CK2, MLC, and Tpm 3.1 bind to actin rings. Mical3 and septins are present at the AIS and control the AIS structure.

Neurons generally possess several primary features. The cell body contains the nucleus and gives rise to two types of processes: axons and dendrites. Axons are the action potential (AP)-transmitting element of neurons, and the AP is initiated at the initial segment of the axon (AIS) and propagates to the synapse. In myelinated axons, the AP is regenerated at the nodes of Ranvier, with small gaps in the myelin sheath. Na^+^ channels are located at high density in the AIS, as well as at the nodes of Ranvier (Figure 1). In addition to regulating AP initiation, the AIS maintains axon-dendrite polarity [1,2,3,4]. To focus on the physiological and pathological excitability-dependent plastic change of the AIS structure, we mainly emphasize the role of the AIS in AP initiation in this review rather than polarity regulation.

The AIS of a neuron comprises the proximal 20–60 μm at the interface between the axon and cell body. Palay and Peters first described the characteristic structures using electron microscopy [5,6]. The internal structure of the AIS is characterized by specific features segregated from the axon hillock and neuronal cell body. Interestingly, different types of neurons have distinct AIS locations, AIS lengths, and ion channel properties [7,8]. It is well accepted that the density of the Na^+^ channels is higher in the AIS than in the body. The AIS is not only the beginning of the axon, but also a key domain in the control of neuronal excitability [9,10,11].

In this review, we first summarize the molecular, structural, ion-channel characteristics, and cell−cell interactions of the AIS. We then describe the activity-dependent plasticity of AIS, primarily by developmental activity. To link the physiological and pathological roles of the AIS in neurodevelopmental disorders (NDDs), we investigate recent advances in human mutation studies of AIS regulatory genes that are highly related to NDDs. The anatomical properties of AIS can be plastic in response to pathological activity; therefore, we discuss recently discovered abnormal pathophysiological changes in the AIS in NDD-model animals and human patients with NDDs. Future studies of the AIS may provide potentially valuable tools for determining the structural and functional neuronal plasticity in response to abnormal network activity in vivo.

## 2. Molecular Characteristics of the AIS

The AIS, 20–60 μm in length, is located at the proximal interface between the axon and cell body. Many studies have shown that the AIS contains a wide array of proteins, including microtubules and the plasma membrane (Figure 1) [1,2,3,4]. Among these, ankyrin-G (ANK3), a neuron-specific protein located at the AIS and nodes of Ranvier, is thought to be the most critical component of the scaffold structure of the AIS.

Ankyrin-G has a membrane-binding domain at the N-terminus, as well as a spectrin-binding domain, a serine-rich domain, and a C-terminal domain [3]. Ankyrin-G has two main isoforms, 270 and 480 kDa, respectively, that are the major isoforms specifically localized at the AIS and nodes of Ranvier [12,13]. Shortly after axon specification, ankyrin-G begins to cluster, recruiting AIS components, such as spectrin tetramers, at the proximal axon [14,15]. Most AIS protein components interact with ankyrin-G either directly or indirectly, and knockdown or knockout of ankyrin-G completely disrupts the localization of AIS proteins [16,17,18]. Therefore, ankyrin-G has been proposed as a master regulator of the AIS structure and polarity [1,2,3,4].

In addition to ankyrin-G, spectrins are essential components of the AIS structure. Spectrin tetramers consisting of two α and two β subunits are widely expressed in neurons. Vertebrate axons possess α-spectrin and five types of β spectrins: βI–βV [19]. Spectrin tetramers contain the βIV spectrin subunit at the AIS and nodes of Ranvier (Figure 1) [20]. The 280 kDa full-length isoform of βIV-spectrin contains an actin-binding domain, 17 triple-helical spectrin repeats, and a specific pleckstrin-homology domain [3]. Recently, the αII-spectrin subunit has been identified as the partner of the βIV-spectrin at the AIS and nodes of Ranvier [21,22]. Spectrin tetramers link adjacent actin rings, as shown in Figure 1. Knockout of αII-spectrin results in embryonic lethality as well as cardiac, craniofacial, and neural tube malformations in the embryo [23]. The conditional deletion of αII-spectrin leads to the disruption of the AIS structure; therefore, αII-spectrin is also an important AIS protein, even though it is expressed throughout neurons, rather than only in the AIS [22,24]. Similarly, the loss of βIV-spectrin in the brain induces disruption of the AIS structure in vivo [25]. 

This modular structure allows ankyrin-G to organize the AIS scaffold (Figure 1) [3]. It anchors AIS-specific membrane proteins, including voltage-gated sodium (Nav) and potassium (Kv) channels, initiating AP (Figure 1). Ankyrin-G knockdown prevented the localization and clustering of AIS proteins, including sodium channels [16,17,18]. 

As described in Section 4 below, the major subtypes of Na^+^ channels at the AIS are Nav 1.1, 1.2, and 1.6 [9,10,11]. In addition, K^+^ channels at the AIS are also important for modulating the AP. Kv7.2 and Kv7.3 (KCNQ2/3) channels can form homomeric or heteromeric complexes and accumulate at the AIS without binding to ankyrin-G [9,10,11].

Conversely, the mechanism of ankyrin-G clustering at the AIS is strongly related to the complex of Ankyrin-B, αII-spectrin, and βII-spectrin. This complex excludes ankyrin-G from the distal axon, confining it to the proximal axon [15]. Neuronal polarity and the developmental process of the AIS is well discussed in an excellent review [26]. The dynein regulator Ndel1 was also stably anchored at the AIS by its interaction with ankyrin-G. Ndel1 activates the retrieval of vesicles transported by dynein in the AIS [27]. 

Additionally, two cell adhesion molecules, neurofascin 186 (NF-186) and NrCAM, were found to exist in the AIS; however, an initial study showed that these adhesion molecules are not necessary for Nav channel clustering [18,28]. Recent studies have also shown that NF-186 is not required for AIS assembly, but is required for AIS maintenance in vivo [29]. Other studies have also shown that NF-186 knockdown induces AIS structural abnormalities [30,31]. Therefore, further investigation is necessary to fully understand the molecular functions of NF-186.

Among microtubule-associated proteins, the C-terminus of ankyrin-G connects to microtubules via interactions with EB1/3. EB3 knockdown alters Nav localization [32]. TRIM46 was shown to be uniquely enriched in the proximal AIS and does not regulate AIS maintenance [33] (Figure 1). Cerebellar Purkinje cell-specific microtubule cross-linking factor 1 (MTCL1) knockout mice exhibit the loss of axonal polarity and mislocalization of ankyrin-G [34].

In addition to these proteins, several other adhesion molecules, such as Tag1, Caspr2, and disintegrin and metalloproteinase domain-containing protein 22 (ADAM22), have been shown to localize in the distal AIS, although no knockout mice studies of these molecules have demonstrated disruption of the AIS compartment [35]. 

Among actin-associated proteins, CK2 (Casein kinase2), the actin ring-related proteins, myosin light chain (MLC), and tropomyosin (Tpm) 3.1 are important for AIS structural maintenance [36,37,38]. Recent studies have also shown that Mical3 (microtubule associated monooxygenase, calponin, and LIM domain containing 3), an oxidoreductase that depolymerizes F-actin, colocalizes with spectrins in the insoluble fraction of AIS, regulating the AIS assembly transiently and via actin patches [39]. The same research team also showed that septins, small GTP binding proteins, are also localized in the insoluble fraction of the AIS, and that knockdown of septins induces the disruption of the AIS assembly [39]. 

Ultimately, these results show that regulatory proteins for microtubules and the actin cytoskeleton and AIS-specific components, such as ankyrin-G or spectrins, are all important components in the regulation of the structure of the AIS.

## 3. Structural Characteristics of the AIS

Palay and Peters initially described the characteristic structure of the AIS using electron microscopy [5,6]. The internal structure of the AIS is characterized by three special features: (*a*) scattered clusters of ribosomes, (*b*) a dense layer of fine granular material beneath the plasma membrane, and (*c*) fascicles of microtubules. 

Subsequent investigations using a super-resolution imaging method, called stochastic optical reconstruction microscopy (STORM), have shown the unique structure of the axon [40]. Actin forms ring-like structures wrapped around the surface of the axons. These rings are evenly spaced every 190 nm. This structure is not observed in dendrites [40]. In the AIS, a periodic assembly of actin rings, βIV-spectrin, and ankyrin-G have been observed [41,42]. 

Simultaneously, the actin structure in the AIS has been shown using platinum replica electron microscopy (PREM). In the center of the AIS cytoskeleton exist bundles of microtubules coated with a dense, fibrillar–globular actin [43]. Immunogold PREM has shown that the actin coat contains AIS proteins such as neurofascin, NrCAM, or Nav [43]. 

Finally, a 2019 study by Vassilopoulos et al. unroofed the dorsal part of cultured neurons and used PREM and STORM to observe that the actin rings are likely to be in the form of twisted ropes containing two long, intertwined actin filaments connected by a dense mesh of aligned spectrins [44]. As discussed in their 2021 review article, similar techniques conducted without unroofing the dorsal part of neurons will help us to understand the structure of whole actin rings at the ultrastructural level [45].

## 4. Ion Channel Properties of the AIS

Physiological studies have shown direct evidence that APs are initiated at the distal end of the AIS in many types of neurons [9,10,11]. It has also become clear that the AIS is not simply a trigger zone for AP generation but also plays a key role in regulating the integration of synaptic inputs, intrinsic excitability, and transmitter release, as has been intensively discussed [9,10,11]. 

Na^+^ channels are involved in the rapid depolarization of APs. Of the four Na^+^ channel α-subunits (Nav 1.1, Nav 1.2, Nav 1.3, and Nav 1.6) expressed in the brain, three subtypes (Nav 1.1, Nav 1.2, and Nav 1.6) are localized in the AIS according to their developmental diversity. Immunocytochemical studies have shown that the major Na^+^ channel isoform in the AIS of adult CNS neurons is Nav 1.6 [46,47]. Interestingly, a tight coupling of Na^+^ channels to the actin cytoskeleton was found to prevent access to the Na^+^ channels in the AIS by the patch-clamp technique [48]. Furthermore, a method was established to unbiasedly match the properties of a wide range of APs in a morphologically realistic model to accurately determine the distribution of Na^+^ channels [48]. Using this approach, the number of Na^+^ channels in the hippocampal pyramidal neuronal AIS was estimated to be 50 times greater than that in the cell body [48], but only five times greater than that in dentate granule cells [49]. In addition to their high density, the properties of Na^+^ channels in the AIS are unique, probably because they facilitate the initiation of APs in the AIS [9,10,11]. 

K^+^ channels are essential for AP repolarization and play a role in setting the AP threshold, interspike intervals, and firing frequencies [9,10,11]. The predominant K^+^ channel in the AIS of most neurons is the low-threshold Kv1 subtype [9,10,11]. Most neurons possess both Kv 1.1 and Kv 1.2 in the AIS [9,10,11]. Direct patch-clamp recording from the AIS showed a high density of dendrotoxin (DTX)-sensitive, fast-activating, slow-inactivating Kv1-type K^+^ currents in the AIS of cortical pyramidal neurons [9,10,11]. In addition, the distal part of the AIS of pyramidal neurons contains a high density of Kv 7.2 and Kv 7.3 (KCNQ2/3) channels [9,10,11].

Taken together, these data indicate that the expression patterns of different ion channels in the AIS are highly cell-specific.

## 5. Non-Cell-Autonomous AIS Regulation through Axo-Axonic Synapse and Axo-Glial Interactions

To the best of our knowledge, the only neurotransmission at the AIS through ligand-gated ion channels uses γ-aminobutyric acid type A receptors (GABA_A_Rs) [50]. GABA_A_Rs containing α2 subunits (α2-GABA_A_Rs) are specifically enriched in the AIS [50]. The AISs of the pyramidal cells of the forebrain contain inhibitory synapses that are exclusively innervated by chandelier cells (ChCs) [51,52]. Hines et al. showed that the localization of α2-GABA_A_Rs to the AIS is essential for the inhibitory control of pathological excitation [53].

The axons of ChCs are highly branched and characterized by arrays of vertically oriented terminals called cartridges, each of which holds synaptic connections [54]. Tai et al. found that the L1 family member L1CAM is required for ChC AIS innervation and maintenance [55]. Selective innervation of the pyramidal neuron AIS by ChCs requires the anchoring of L1CAM to the AIS by the cytoskeletal ankyrin-G/βIV-spectrin complex [54]. 

More recently, Pan-Vazquez et al. found, by in vivo imaging, that ChC axons and their axo-axonic synapses develop rapidly in infant mice from postnatal day (P)12 to P18 [56]. They also found that an increasing network activity during this period reduces the number of axo-axonic synapses and that the AIS length changes slightly [56]. In older mice (P40–P46), when ChC synapses switch to inhibitory, they result in an increase in axo-axonic synapses. The depolarizing nature of axo-axonic synapses suggests that this plasticity is homeostatic. In addition, increasing ChC activity may decrease the AIS length of pyramidal neurons that are connected to activated ChCs [56].

In glial cells, Rasband et al. reported an interesting interaction between microglia and the AIS [57]. Microglia are immune cells that reside in the brain and are actively involved in the regulation of neuronal excitability and function. Rasband et al. found that in the cerebral cortex, a subset of microglia spatially extended a single process that binds to the AIS. The interaction between microglia and AISs appears early in development and persists into adulthood. However, these interactions are reduced after brain injury due to the activation of microglia [57]. Recently, in a hypoglossal nerve injury model, Tamada et al. observed microglia—AIS interaction and the accumulation of mitochondria in the AIS after injury [58].

Astrocytes, the most abundant cells in the CNS, promote synapse formation and help refine neural connectivity. Molofsky et al. showed that the loss of astrocyte-encoded semaphorin Sema-3a leads to a dysregulated α-motor neuron AIS orientation [59]. 

Lastly, regarding oligodendrocytes, cuprizone treatment increases axonal excitability in dysmyelinated mouse brains. Membrane repolarization and energy expenditure may be affected by the general misalignment of ion channels in the AIS [60]. The AIS of demyelinated axons begins closer to the cell body than in myelinated axons, but the expression of ankyrin G, βIV-spectrin, and ion channels was maintained [60]. Another study replicated this result using a genetic demyelination model [61]. These results suggest that myelination by oligodendrocytes is important for the maintenance of the AIS by the inhibition of the hyperexcitability of pyramidal neurons.

Taken together, these results indicate that the AIS can be controlled either in a cell-autonomous fashion or in a non-cell-autonomous fashion.

## 6. Activity-Responding Plasticity of AIS in Development and Disease Models

Different types of neurons differ in the location, length, and ion channel configuration of the AIS [1,2,11,62]. Recent studies have shown that the structural properties of the AIS are plastic in response to normal developmental and pathological activities [1,2,11,62]. Since other rapid inhibitions of electrical properties of the neuronal AIS in vitro are well- documented [63,64], this review focuses on the plastic changes of the AIS in vivo.

Kuba et al. strikingly showed that the AIS in chicken nucleus magnocellularis neurons increased after deprivation of auditory inputs and moved closer to the cell body [65]. Physiological recordings also showed that these cells become more excitable [65]. The same research team recently reported that the regulation of cytoskeletal reorganization and sodium channel enrichment in the AIS differ depending on tonotopy (the spatial arrangement of sound frequency processing in the brain), but act synergistically in the auditory nucleus [66]. These results indicate that neurons adapt to presynaptic activity and maintain neural circuit homeostasis by altering the properties of the AIS. 

Expanding evidence of the structural changes and remodeling of the AIS has been observed in disease models, such as type 2 diabetes [67], epilepsy [68], cerebral infarction [69], neuroinflammation by LPS [70], traumatic brain injury [71], Alzheimer’s disease [72], amyotrophic lateral sclerosis [73] and mutations in the *Tau* gene causing frontotemporal dementia [74]. We will discuss these observations at length in Section 8.

In addition to these studies, cortical neurons undergo dynamic changes in the AIS in the visual cortex during the critical period of visual system development [75]. However, these changes were prevented by visual deprivation for postnatal weeks [75]. Gutzmann et al. also showed the relationship between the cisternal organelle (CO) and the AIS during visual cortex development. Synaptopodin-deficient mice lack COs and showed a shortening of the AIS in the dark for three to five weeks [76]. Another study showed an observed structural plasticity of the AIS in response to various auditory experiences at varying mouse ages [77].

More interestingly, long-term sensory deprivation altered the AIS length of layer II/III pyramidal neurons in the sensory cortex, and the same cortical neurons rapidly decreased the AIS length in an enriched environment [78]. In addition to visual and somatosensory deprivation, Galliano et al. showed that odor deprivation by naris occlusion produced AIS shortening and a decrease in intrinsic excitability in axon-bearing dopaminergic neurons in the glomerular layer of olfactory bulbs [79]. 

Taken together, these results suggest that bidirectional activity-dependent remodeling of the AIS by various types of sensory inputs plays a role in homeostatic adaptation in vivo.

## 7. Association and Mutation Studies on AIS-Related Genes

Molecular studies have shown the importance of AIS proteins, and clinical studies have shown that mutations in AIS regulatory genes are highly related to the etiology of neurodevelopmental disorders. 

In general, the Diagnostic and Statistical Manual of Mental Disorders (DSM-5) states that neurodevelopmental disorders (NDDs) include intellectual disability (ID), communication disorders, autism spectrum disorder (ASD), attention deficit hyperactivity disorder (ADHD), specific learning disorder, and motor disorders [80]. However, NDDs are complex conditions [81]; therefore, this review broadly covers various classes of central nervous system (CNS) disorders that manifest during development, such as epilepsy, mood disorders, and schizophrenia. 

Among the many AIS-localized proteins, as shown in Table 1, we focus on three genes: *ANK-3*, which encodes human ankyrin-3 protein (also known as ankyrin-G), *SPTAN1*, which encodes spectrin alpha chain, non-erythrocytic 1 protein, and *SPTBN4*, which encodes spectrin beta chain, non-erythrocytic 4 protein. Ion channel mutations that cause channelopathy are well described in excellent reviews [82,83].

In patients with bipolar disorder (BD), several studies have shown that *ANK-3* has strong predisposition to BD in a genome-wide association study [84,85,86]. Exon variation has also been intensively investigated in patients with BD. Interestingly, exon variation at a BD-associated SNP correlates with a significant difference in the cerebellar expression of a brain-specific *ANK-3* transcript and contributes to disease pathology [87,88]. *ANK-3* is also associated with schizophrenia [89,90,91]. 

Recently, whole-exome sequencing allowed the identification of a pathogenic mutation in the *ANK-3* gene of patients with ASD (Table 1) [92]. In addition, another mutation has been identified within a family of patients with ASD [93]. Iqbal et al. reported multiple types of *ANK-3* mutations in patients with NDDs [94]: one patient had borderline intelligence, ADHD, ASD, and cognitive problems caused by the balanced chromosomal translocation of *ANK-3*, and in a Pakistani family, moderate ID, and ADHD-like phenotype and behavioral problems were associated with a homozygous single base pair truncating frameshift mutation in *ANK-3* [94]. Subsequently, several *ANK-3* mutations were found in patients with ID [95,96,97,98]. More recently, two patients with NDDs and *ANK-3* mutations were identified; each exhibited mild-to-borderline ID, ASD-like features, and speech delays. One patient also exhibited developmental delays, seizures, cognitive impairment, ataxia, retinal dystrophy, and small stature [99]. 

The first pathogenic variant was found in *SPTAN1* in patients with West syndrome, a constellation of symptoms primarily characterized by epileptic/infantile spasms, abnormal brain electroencephalography (EEG) patterns called hypsarrhythmia, and ID (Table 1); two patients possessed a heterozygous in-frame 3bp deletion or 6bp duplication in *SPTAN1* [100]. The patients exhibited intractable seizures at three months of age and showed ID, poor visual attention, a lack of speech development, and spastic quadriplegia; in addition, brain magnetic resonance imaging (MRI) showed diffuse hypomyelination and extensive brain atrophy. Subsequently, several studies have also identified a mutation in *SPTAN1* in patients with a wide spectrum of neurodevelopmental phenotypes ranging from mild to severe (Table 1) [101,102,103,104].

In addition, four heterozygous mutations in *SPTAN1* were recently identified in four different families with juvenile-onset hereditary motor neuropathy, a particularly rare subgroup of inherited peripheral neuropathies [105,106]. These patients typically exhibited a length-dependent axonal degeneration of lower motor neurons without the apparent involvement of sensory neurons [105,106]. 

Recently, the first pathogenic variant of *SPTBN4* was reported [107]. Other studies have also shown mutations in *SPTBN4* [108,109,110,111,112]. These congenital diseases are defined as SPTBN4 disorder or, alternatively, neurodevelopmental disorder with hypotonia, neuropathy, and deafness (NEDHND) [113]. 

These results indicate that mutations in these AIS regulatory proteins are important to the development of the CNS.

## 8. Abnormal AIS Characteristics in Neurodevelopmental Disorders

Studies on human mutations have shown that molecules that regulate the AIS are highly related to brain development. Finally, we discuss the changes in the properties of the AIS in animal models and patients with NDDs, as well as the potential use of homeostatic regulation for the treatment of NDDs.

The first report by Kaphzan et al. showed a significant increase in the AIS length of hippocampal pyramidal neurons in vivo in an Angelman syndrome (AS) mouse model [114]. AS is an NDD caused by a loss of function of the maternally inherited *UBE3A* gene and is associated with symptoms of ID, epilepsy, cerebellar ataxia, sleep disorders; AS also shows high comorbidity with ASD. Changes were also found in the amplitude of the resting membrane potential, threshold potential, and action potential [114]. Interestingly, CA1 and CA3 pyramidal neurons have a significantly longer AIS, whereas cortical neurons in the somatosensory area do not show alterations [114]. This is the first in vivo evidence of changes in the AIS in an animal model of NDDs [114].

As mentioned earlier, a mutation in the *ANK-3* gene, which encodes the 480 kDa ankyrin-G isomer, induces NDDs in humans [99]. In addition, disruption of exon 37 induces a specific giant ankyrin-G disruption, and exon 37 knockout mice showed reduced cortical gamma oscillations, which are associated with higher cognitive processes [99]. Further behavioral investigations of exon 37 knockout mouse or new animal models of AIS protein with human disease mutation, may strongly support the use of the mouse as an ideal model animal for NDDs.

More recently, in a mouse model of the fragile X syndrome, Booker et al. showed that the length of the AIS in CA1 neurons in the Fmr1^−/y^ mouse hippocampus elongated with increasing cellular excitability. This change in length was not due to a decrease in AIS plasticity but instead to a reduced input from the entorhinal cortex. These results indicate that the length of the AIS and cellular intrinsic hyperexcitability may reflect a decrease in the synaptic input to CA1 neurons in a non-cell-autonomous fashion by homeostatic mechanisms [115].

In humans, Hong et al. showed that the α2-GABA_A_R protein is reduced in the AIS of pyramidal cells in the prefrontal cortex in patients with ASD [116]. They first showed a significant decrease in the number of ChCs in the prefrontal cortex of patients with ASD [117], and that the reduction in α2-GABA_A_R in the pyramidal cell AIS was localized to layer III in ASD patients, presumably due to ChC reduction [116]. These results support the idea that ChCs regulate the cortical excitability of pyramidal neurons in the AIS domain.

Another study found that, in mouse models of ASD, mice with mutations in the transcription factor PAX6 exhibited changes of the AIS that were significantly further from the cell body and exhibited a longer AnkG staining of prethalamic neurons [118]. 

Taken together, these results suggest that the AIS is an indicator of structural changes as well as abnormal and homeostatic plastic changes in response to the dysfunction of neural circuits.

## 9. Conclusions

As discussed above, a growing body of evidence suggests the importance of the pathophysiological role of abnormal AISs in NDDs. This then prompts the question: can we take advantage of these concepts to develop future treatments or diagnostic tools based on AIS changes? One of the most promising uses of the AIS is as a biomarker for aberrant neural circuits in neurological and psychiatric disorders. In a variety of neurological and psychiatric disease models, an abnormal AIS length has been widely observed [68,69,70,71,72,73,74,114,115]. To determine cell-specific neural circuit abnormalities, researchers can use viral or classical retrograde or anterograde tracing methods in disease models. Then, chemogenetic or optogenetic techniques can be applied to modulate circuit activity and prove the aberrant circuits responsible for disease. 

On the other hand, we may also ask: are there any potential therapies for NDDs? According to an excellent review from 2021, we now have several future opportunities to manage NDDs therapeutically at genome, protein, neural circuit, and individual levels using recent technical advances such as CRISPR-Cas9, optogenetics, chemogenetics, the transplantation of differentiated neurons, and various brain stimulation methods [119]. 

In particular, according to the results of a human study on ChCs in ASD [116], ChCs may be decreased in the ASD brain; therefore, it may be useful to transplant human ChCs that are differentiated from pluripotent cells into patients with ASD [119]. 

Recent advances in ultrasensitive optogenetics may allow the use of implant-free deep brain optogenetics that could be used for human neural circuit-specific therapy [120,121]. If abnormal neural circuits were determined in disease models, human neural circuits could be modulated by the viral vector with optogenetic methods, even deep in the brain. 

## Figures and Tables

**Table 1:** Mutations of *ANK-3*, *SPTAN1*, *SPTBN4*, and related symptoms.

Gene Name Accession # (Chromosome #)	Gene Mutation Locus	Mutated Protein	Symptoms	References
ANK-3 NM_020987.3 (10q21)	c.4705T > G c.11159C > T * c.12763A > C * c.11068G > A 46,XY,t(2;10)(q11.2;q21.2) c.10995delC c.9652C > T c.1990G > T c.11033del c.4960G > T, c.4465C > T	p.S1569A p.T3720M p.T4255P p.G3690R p.T3666LfsX2 p.L3218F p.G664 * p.P3678Lfs *45 p.D1654Y, p.P1489S	ASD ADHD, ASD ID, hypotonia, spasticity ID, brain atrophy, delayed myelination ID, ASD, macrocephaly ID Severe intractable seizures with DD	Bi et al., 2012 Shi et al., 2013 Iqbal et al., 2013 Karaca et al., 2015 Kloth et al., 2017 Hu et al., 2019 Zhu et al., 2020
SPTAN1 (SPECTRIN, ALPHAII) NM_0011304 (9q34.11)	c.6619-6621del c.6923-6928dup c.1697G > C c.6605-6607del c.6908-6916 dup c.6910_6918 dup c.5326C > T c.6184C > T c.6619_6621del c.6619_6621del c.6622_6624del c.6811G>A c.6850_6852del c.6908_6916dup c.6908_6916dup c.6908_6916dup c.6907_6915dup c.6907_6915dup c.6910_6918del c.6923_6928dup c.533G > A c.917C > T c.3716A > G c.4828C > T c.6908_6916del arr[hg19] 9q34.11 (131,349,701–131,351,531) x1 exon 20–21 deletion heterozygous c.415C4T heterozygous c.4615C4T heterozygous c.6385C4T heterozygous c.6781C4T	p.E2207del p.R2308_M2309dup p.R566P p.Q2202del p.D2303_L2305dup p.Q2304_G2306dup p.R1776W p.R2062W p.E2207del p.E2207del p.N2208del p.E2271K p.D2284del p.D2303_2305dup p.D2303_2305dup p.D2303_2305dup p.D2303_2305dup p.D2303_2305dup p.Q2304_G2306del p.R2308_M2309dup p.G178D p.A306V p.H1239R p.R1610W p.D2303_L2305del p.A927_L1002del p.R139 * p.Q1539 * p.Q2149 * p.R2261 *	WS, profound ID, spastic quadriplegia Mild ID, IS WS, severely impaired psychomotor development WS Infantile EE with IS and focal epilepsy, mild ID, ASD Infantile EE with tonic spasms and FDS, profound DD, severe hypotonia, microcephaly WS, profound DD, minimal interaction, hypotonia, hypokinesia; microcephaly WS, profound DD, hypotonia, microcephaly WS, profound DD, severe hypotonia, thermic dysregulation; microcephaly WS, profound DD, hypotonia, multifocal myoclonus, dyskinetic movement disorder, microcephaly Infantile EE with IS evolving to myoclonic seizures, severe DD, hypotonia, ataxic movement disorder WS, profound DD, hypotonia, ataxia, dyskinetic movement disorder, microcephaly WS (PEHO syndrome), profound DD, hypotonia, microcephaly WS, profound DD, hypotonia, microcephaly WS, profound DD, hypotonia, ataxia, dyskinetic movement disorder, microcephaly WS, profound DD, intermittent opisthotonus, hypotonia, microcephaly WS, mild ID, DD, delayed walking WS, profound DD, microcephaly Focal epilepsy Epilepsy with myoclonic and atonic seizures, moderate ID No epilepsy, mild DD, ID, ASD; mild dysmorphic signs Myoclonic epilepsy, mild–moderate DD, ID, ASD, hypotonia, mild spastic gait; hyperreflexia in lower limbs FDS, moderate ID, ADHD Focal seizures, mild DD, ID, mild diffuse hypotonia, slowly progressive and severe cerebellar ataxia Hereditary motor neuropathy	Saitsu et al., 2010 Hamdan et al., 2012 Nonoda et al., 2013 Ream and Mikati 2014 Syrbe et al., 2017 Beijer et al., 2019 Dong et al., 2021
SPTBN4 (SPECTRIN, BETAIV) NM_020971.2 (19q13.2)	homozygous c.1597C > T homozygous c.3394del homozygous c.3820G > T homozygous c.2709G > A c.1511G > A; c.7303C > T homozygous c.7453del c.1813C > T; c.3829del homozygous c.1665+2T > C homozygous c.3949-1G > A	p.Q533 * p.H1132Tfs *39 p.E1274 * p.W903 * p.R504Q; p.R2435C p.A2485Lfs *31 p.Q605 *, p.Q1277Rfs *4 Intron Intron	Congenital myopathy, neuropathy, and central deafness Global DD, hypotonia, dysphasia, recurrent respiratory infections, blue sclerae, hyporeflexia Profound ID, congenital hypotonia, and motor axonal neuropathy Speech delay, ID, ataxia, seizures, cerebral atrophy Axonal neuropathy without ID	Knierim et al., 2017 Anazi et al., 2017 Wang et al., 2019 Monies et al., 2019 Hausler et al., 2020

Abbreviations: number (#); termination codon (*); frame shift (fs); duplication (dup); deletion (del); autism spectrum disorder (ASD); attention deficit hyperactivity disorder (ADHD); intellectual disability (ID); West syndrome (WS); developmental delay (DD); epileptic encephalopathy (EE); infantile spasms (IS); focal dyscognitive seizures (FDS); progressive encephalopathy (PEHO).

## Data Availability

No new data were created or analyzed in this study. Data sharing is not applicable to this article.
